# Mesial–Distal Patterns of Alveolar Bone Loss in Periodontitis and Peri-Implantitis: A Comparative Radiographic Study

**DOI:** 10.3390/dj14090546

**Published:** 2026-09-01

**Authors:** Efstathios Grammatikis, Corina Laura Ștefănescu, Liliana Sachelarie, Marius-Florentin Popa, Marius-Daniel Radu, Rodica-Maria Murineanu, Elena Șapte

**Affiliations:** 1 Doctoral School, Faculty of Dentistry, Ovidius University, 900527 Constanta, Romania; stathisgrammatikis@icloud.com; 2 Faculty of Dentistry, Ovidius University, 900527 Constanta, Romania; corina.stefanescu@365.univ-ovidius.ro (C.L.Ș.); rodica.murineanu@365.univ-ovidius.ro (R.-M.M.); 3 Department of Dental Medicine, Apollonia University, 700511 Iasi, Romania; 4 Preclinical Department 1, Faculty of Medicine, Ovidius University, 900527 Constanta, Romania; 5 Faculty of Natural and Agricultural Sciences, Ovidius University, University Alley, No. 1, Campus, Building B, 900470 Constanta, Romania; marius.radu@univ-ovidius.ro; 6 Discipline of Anatomy, Faculty of Dentistry, Ovidius University, 900527 Constanta, Romania; esapte@univ-ovidius.ro

**Keywords:** periodontitis, peri-implantitis, alveolar bone loss, marginal bone loss, radiographic assessment, mesial bone loss, distal bone loss, dental implants

## Abstract

**Background/Objectives**: Periodontitis and peri-implantitis are inflammatory conditions associated with progressive loss of supporting bone, but they differ in tissue architecture, biological response, and disease progression. Conventional radiographic assessment often reports bone loss as a global value, which may overlook site-specific mesial–distal differences. This study aimed to compare mesial and distal radiographic bone loss around natural teeth affected by periodontitis and dental implants affected by peri-implantitis, with a focus on quadrant 1. **Methods**: This retrospective comparative CBCT-based radiographic study analyzed 128 within-patient paired tooth/implant observations in quadrant 1. Mesial and distal bone loss values were recorded separately for natural teeth and corresponding implant sites. Each pair included one natural-tooth site and one implant site from the same patient, matched by comparable anatomical position and surface. Paired-samples *t*-tests were used, and mean differences were calculated as implants minus natural teeth. Given the multiple site-specific comparisons, *p*-values were interpreted as unadjusted exploratory findings. **Results**: Statistically significant differences were observed only on the mesial surface of anterior implants. Mesial bone loss was significantly greater around implants than natural teeth for central incisors (8.30 ± 3.31 mm vs. 4.90 ± 1.78 mm; mean difference = 3.39 mm; *p* = 0.004), lateral incisors (8.12 ± 3.57 mm vs. 4.94 ± 2.82 mm; mean difference = 3.18 mm; *p* = 0.028), and canines (5.99 ± 3.12 mm vs. 4.42 ± 1.26 mm; mean difference = 1.57 mm; *p* = 0.018). No statistically significant differences were observed for distal surfaces or posterior tooth/implant positions. **Conclusions**: Within the limitations of this quadrant-specific retrospective radiographic analysis, peri-implant bone loss showed a surface- and position-dependent distribution, with greater mesial bone loss around anterior implants. Separate mesial and distal assessment may provide additional value for periodontal and peri-implant monitoring.

## 1. Introduction

Periodontitis and peri-implantitis are two inflammatory conditions characterised by progressive destruction of the supporting tissues around natural teeth and dental implants, respectively. Although they share several clinical similarities, including inflammation, pocket formation, bleeding on probing, and radiographic bone loss, they differ in tissue architecture, biological response, and disease progression patterns [1,2,3,4,5,6]. The 2017 World Workshop classification introduced a unified framework for periodontal and peri-implant diseases, emphasizing the need for precise diagnostic criteria and standardized evaluation of tissue destruction around both teeth and implants [1,2,3,4,5]. Periodontitis is currently defined within a staging and grading system that reflects severity, complexity, extent, and progression risk, while peri-implantitis is characterized by inflammation of the peri-implant mucosa associated with progressive loss of supporting bone [2,3,4,5,6,7].

Alveolar bone loss remains one of the most relevant indicators for assessing the severity and progression of periodontal disease. Around natural teeth, bone loss is commonly evaluated radiographically by measuring the distance between the cementoenamel junction and the alveolar bone crest, particularly on mesial and distal surfaces [8,9,10,11]. In implant dentistry, marginal bone level changes are usually assessed by measuring the distance from a fixed implant reference point to the first radiographic bone-to-implant contact, also most frequently on the mesial and distal aspects [12,13]. These measurements are clinically important because they allow for objective monitoring of bone support and may help identify early disease progression before severe clinical complications occur.

Despite apparent similarities, peri-implantitis should not be considered a simple equivalent of periodontitis around an implant. Natural teeth are surrounded by periodontal ligament, cementum, and a highly organized connective tissue attachment, while dental implants are anchored directly to bone through osseointegration. These anatomical and biological differences may influence the pattern, speed, and morphology of bone loss [6,9,10].

Previous histopathological and contemporary review evidence has shown that peri-implantitis lesions may be larger and contain a more extensive inflammatory infiltrate than periodontitis lesions, suggesting that peri-implant tissue breakdown may follow a distinct biological pathway [9,10]. Radiographic assessment has an essential role in both periodontal and peri-implant diagnosis.

In periodontitis, mesial and distal bone levels can reflect the extent of interproximal alveolar destruction, while in peri-implantitis, mesial and distal marginal bone loss may provide important information regarding implant prognosis and treatment planning [11,12,13].

However, previous radiographic studies commonly assessed periodontal and peri-implant bone loss using marginal bone-level measurements [11,12,13]. Although mesial and distal bone levels are routinely evaluated radiographically [11,12,13], the resulting measurements are frequently summarized as mean or overall bone-loss estimates, while the measurements from mesial and distal surfaces are less frequently retained as distinct site-specific comparative outcomes [1,2,3]. Moreover, although periodontal and peri-implant bone destruction have been extensively characterized [6,9,10], direct within-patient comparisons according to anatomical position and individual interproximal surface remain limited. Consequently, global or averaged measurements may obscure localized or asymmetric patterns of bone destruction, particularly when disease progression is not uniformly distributed around a tooth or implant [1,2,3].

A more detailed topographic analysis of bone resorption may be clinically relevant, particularly when comparing natural teeth affected by periodontitis with dental implants affected by peri-implantitis. Mesial and distal surfaces may be differently influenced by anatomical conditions, prosthetic design, implant positioning, occlusal loading, hygiene accessibility, and local inflammatory response. Therefore, separate evaluation of these surfaces may offer additional information beyond conventional global bone loss assessment.

From a clinical perspective, identifying whether bone loss is predominantly mesial or distal may help detect localized disease progression that could be obscured by global or averaged measurements. Such site-specific information may prompt closer evaluation of local contributing factors, including implant positioning, prosthetic contour, hygiene accessibility, and local anatomical conditions, and may support individualized maintenance and treatment planning. Moreover, early recognition of a surface-specific pattern of bone loss may contribute to more accurate assessment of disease progression and prognosis.

In this context, the present study aims to compare alveolar bone resorption around natural teeth affected by periodontal disease and dental implants affected by peri-implantitis, using a mesial–distal radiographic approach. By analyzing bone loss according to tooth/implant position and surface, this study seeks to identify whether periodontal and peri-implant bone destruction follow similar or distinct radiographic patterns. The working hypothesis is that peri-implantitis and periodontitis may present different site-specific patterns of bone loss, with potentially unequal involvement of mesial and distal surfaces.

The originality of this study lies in its comparative and topographic approach. Instead of evaluating bone loss as a single average, the analysis focuses on the distribution of resorption across mesial and distal surfaces, enabling a more refined comparison between periodontal and peri-implant bone destruction. This approach may contribute to improved radiographic interpretation, earlier detection of localized disease progression, and more individualized periodontal and implant maintenance strategies.

## 2. Materials and Methods

### 2.1. Study Design

This study was designed as a retrospective, comparative, CBCT-based radiographic analysis evaluating alveolar bone resorption around natural teeth affected by periodontal disease and around dental implants affected by peri-implantitis. The study protocol was reviewed and approved by the Institutional Ethics Committee of Ovidius University (Approval No. 622, 13 June 2025). The study was retrospective and based on anonymized CBCT records obtained during routine clinical diagnosis, treatment planning, or follow-up. No additional diagnostic procedures, treatment interventions, or patient contact was performed for the purposes of this research.

The primary objective was to determine whether alveolar bone resorption follows similar or different mesial–distal patterns around natural teeth and dental implants. The secondary objective was to assess whether these differences varied by tooth/implant position and anatomical region within quadrant 1.

The study was based on previously collected radiographic measurements recorded in millimeters for natural teeth and corresponding implant sites. The diagnosis of periodontitis and peri-implantitis had been previously established by the treating clinicians as part of routine clinical care and was documented in the available clinical records, together with radiographic evidence of bone loss. The present analysis focused specifically on the comparative assessment of mesial and distal radiographic bone-level measurements.

The available dataset included separate measurements for mesial and distal surfaces, organized by tooth type and dental quadrant, including incisors, canines, premolars, and molars.

Paired comparisons were performed between corresponding natural tooth and implant measurements, with variable sample sizes depending on the tooth/implant position analyzed.

### 2.2. Study Material and Data Source

The study material consisted of anonymized CBCT-based measurements of alveolar bone loss collected from 128 adult patients presenting periodontal involvement around natural teeth and peri-implant bone loss around dental implants. The records were collected retrospectively from patients evaluated between January 2024 and December 2025.

For each analyzed site, bone resorption was assessed separately on the mesial and distal surfaces. The measurements were organized according to the type of structure evaluated, namely natural teeth affected by periodontitis and dental implants affected by peri-implantitis. The data were further classified according to dental quadrant, tooth or implant position, and the corresponding mesial and distal CBCT bone-level measurements.

The dataset included measurements from both anterior and posterior regions, comprising central incisors, lateral incisors, canines, premolars, and molars. All measurements were expressed in millimeters and were subsequently used for descriptive and comparative statistical analysis.

The final dataset for quadrant 1 comprised 128 within-patient paired tooth/implant CBCT observations distributed across seven anatomical positions. Each paired observation represented a predefined within-patient matched comparison between a natural tooth and a dental implant from the same patient, corresponding to the same anatomical position in maxillary quadrant 1 and the same interproximal surface (mesial or distal). Each patient contributed only one matched tooth–implant pair to the analysis. The number of paired observations varied by tooth/implant position because only sites with complete corresponding natural-tooth and implant measurements were included in each paired comparison.

Specifically, paired observations were available for central incisors (n = 13), lateral incisors (n = 14), canines (n = 22), first premolars (n = 24), second premolars (n = 21), first molars (n = 15), and second molars (n = 19). Therefore, the degrees of freedom differed across the paired-samples *t*-tests.

Because the present analysis was limited to quadrant 1, the findings should be interpreted as quadrant-specific and not automatically generalized to the entire dentition.

As the present study was based exclusively on retrospective analysis of anonymized CBCT records, the available dataset did not include a systematic clinical characterization of the patients. Therefore, smoking status, detailed periodontal history and disease severity, systemic conditions such as diabetes, implant type, and loading characteristics were not evaluated in the present analysis. The available demographic information was limited to sex and age range, while implant location was determined according to anatomical position within quadrant 1.

### 2.3. Inclusion Criteria

Radiographic records were included in the study when they presented natural teeth with periodontal bone loss or dental implants with peri-implant bone loss. Eligible cases had to provide available mesial and distal measurements expressed in millimeters. Only CBCT records/datasets with sufficient image quality were selected to allow for clear identification of anatomical landmarks and implant-related reference points. In addition, sites were included only when complete paired data were available, enabling statistical comparison between natural teeth and corresponding implant sites.

### 2.4. Exclusion Criteria

CBCT records/datasets were excluded if image quality was poor, distortion was present, or reliable assessment of bone level could not be performed. Cases with incomplete measurements on either the mesial or distal surface were also excluded. Additional exclusion criteria included unclear anatomical or implant-related reference points, extensive artifacts interfering with bone level evaluation, and records lacking sufficient information for classification according to dental quadrant or tooth/implant position.

### 2.5. CBCT Assessment and Measurement Protocol

Radiographic bone loss was assessed using cone-beam computed tomography (CBCT) images. CBCT was selected because it allows for three-dimensional visualization of alveolar bone levels around both natural teeth and dental implants and enables separate assessment of mesial and distal surfaces. All CBCT examinations were performed as part of routine clinical diagnosis, treatment planning, or follow-up, and no additional imaging procedures were performed specifically for this study.

CBCT examinations were acquired using a Carestream CS 8200 3D Advance Edition system (Carestream Dental, Atlanta, GA, USA), operating at 60–90 kV and 2–15 mA, with exposure times ranging from 3 to 20 s depending on the selected acquisition protocol. The system provides fields of view ranging from 4 × 4 cm to 16 × 10 cm and a minimum voxel size of 75 μm.

For each examination, the field of view, resolution, and exposure parameters were selected according to the diagnostic objective, the region of interest, and image quality requirements. Whenever possible, the smallest field of view compatible with the area to be assessed was used, in accordance with dose optimization principles. During image acquisition, patients were positioned and stabilized according to the manufacturer’s recommendations in order to reduce movement artifacts and improve the reproducibility of radiographic measurements.

The acquired CBCT data were reconstructed as DICOM datasets and analyzed in multiplanar views. Measurements were performed using the native CBCT viewing software associated with the imaging system, CS 3D Imaging Software, version 3.10.43 (Carestream Dental LLC, Atlanta, GA, USA). Before measurement, each CBCT dataset was checked for image quality, correct orientation, and the absence of artifacts that could interfere with bone-level assessment. The analyzed tooth or implant was evaluated based on the sagittal, coronal, and cross-sectional slices that provided the clearest visualization of the mesial and distal bone levels.

For natural teeth, alveolar bone loss was measured separately on the mesial and distal surfaces as the vertical distance from the cementoenamel junction to the most coronal level of the alveolar bone crest. For dental implants, peri-implant bone loss was measured separately on the mesial and distal surfaces as the vertical distance from the implant shoulder or platform to the first visible bone-to-implant contact. All measurements were performed perpendicular to the reference landmark whenever possible and were recorded in millimeters.

Calibration was performed using the CBCT software’s linear measurement tool. For implant sites, when available, the known implant length and/or diameter was used as an internal reference to verify measurement accuracy. Each measurement was recorded digitally, and mesial and distal values were entered separately into the study database.

A subset of CBCT measurements was repeated by the same examiner after a two-week interval to assess internal consistency. No formal intra-examiner reliability coefficient was calculated. The repeated measurements were compared with the initial values in order to verify reproducibility. When discrepancies were identified, the corresponding CBCT sections were reassessed, and the final value was established after repeated evaluation. Since all measurements were performed by a single calibrated examiner, inter-examiner reliability was not applicable and was considered a methodological limitation.

Mesial and distal values were analyzed separately in order to identify possible asymmetrical patterns of bone loss. This approach allowed for a more detailed, site-specific evaluation of periodontal and peri-implant bone resorption than a global average bone-loss value would have provided.

### 2.6. Variables Analyzed

The main variables analyzed in this study were the mesial and distal bone loss values recorded around natural teeth and dental implants. For natural teeth, alveolar bone resorption was assessed separately on the mesial and distal surfaces. Similarly, for dental implants, peri-implant bone resorption was measured on both the mesial and distal aspects.

Additional variables included the anatomical position of the analyzed tooth or implant, classified as central incisor, lateral incisor, canine, premolar, or molar, as well as the dental quadrant in which the site was located. The difference in bone loss between natural teeth and implants was calculated for each corresponding surface, allowing for a comparative evaluation of mesial and distal resorption patterns.

### 2.7. Comparative Analysis

For each tooth/implant position, mesial and distal bone loss values were compared between natural teeth affected by periodontitis and dental implants affected by peri-implantitis. Comparisons were performed separately for each surface in order to identify possible site-specific differences in bone resorption.

Mesial bone loss around natural teeth was compared with that around implants, whereas distal bone loss around natural teeth was compared with distal peri-implant bone loss. In addition, the analysis considered differences between anterior and posterior regions within quadrant 1.

A paired observation was defined as a natural-tooth site and a dental implant site from the same patient, matched for comparable anatomical position and surface, either mesial or distal. Thus, each paired comparison included two related within-patient measurements, not independent observations obtained from different patients.

This comparative approach was selected to determine whether peri-implant bone loss follows a distribution pattern similar to that of periodontal bone loss around natural teeth or presents a distinct topographic profile. By analyzing bone resorption according to surface, anatomical position, and quadrant, the study aimed to provide a more detailed CBCT-based characterization of periodontal and peri-implant bone destruction.

### 2.8. Statistical Analysis

Data were analyzed using descriptive and inferential statistics. Continuous variables were expressed as the mean, standard deviation, and standard error of the mean. For each paired comparison, mean differences and 95% confidence intervals were calculated.

Because each pair represented a predefined within-patient matched comparison between a natural tooth and a dental implant corresponding to the same anatomical position and interproximal surface, the measurements were treated as related observations and analyzed using paired-samples tests.

Paired-samples *t*-tests were used to compare mesial and distal measurements of corresponding natural teeth and implants. Comparisons were performed separately for each tooth/implant position and surface. Mean differences were calculated as implants minus natural teeth; therefore, positive values indicate greater bone loss around implants. The level of statistical significance was set at *p* < 0.05. The 14 comparisons were predefined a priori according to anatomical position and surface and were not generated through post hoc testing. Given the exploratory nature of this quadrant-specific analysis and the relatively small position-specific subsamples, no formal multiplicity correction was applied to avoid an excessive increase in type II errors. The unadjusted *p*-values were therefore interpreted cautiously alongside effect sizes and 95% confidence intervals.

## 3. Results

### 3.1. General Overview of the Paired Comparative Analysis

The study population comprised 128 patients, including 87 women (68.0%) and 41 men (32.0%), with ages ranging from 45 to 67 years (Table 1).

The radiographic comparative analysis evaluated mesial and distal bone loss around natural teeth and corresponding dental implant sites in quadrant 1. A total of 128 paired tooth/implant observations were included, with variable sample sizes across anatomical positions due to incomplete matching of measurements.

Paired-samples *t*-tests were performed separately for each tooth/implant position and each surface, allowing for site-specific comparisons between periodontal bone loss around natural teeth and peri-implant bone loss around dental implants. The analyzed positions included the central incisor, lateral incisor, canine, first premolar, second premolar, first molar, and second molar.

For each anatomical position, mesial peri-implant bone loss was compared with mesial periodontal bone loss, whereas distal peri-implant bone loss was compared with distal periodontal bone loss. Mean differences were calculated as implants − natural teeth; therefore, positive values indicate greater bone loss around implants.

Overall, statistically significant differences were mainly observed in the anterior region, particularly on the mesial surface. In contrast, distal comparisons and posterior tooth/implant positions did not show statistically significant differences.

The complete descriptive and inferential results of the paired comparisons for quadrant 1 are presented in Table 2, including mean bone loss values, standard deviations, mean differences, t-values, degrees of freedom, and *p*-values for each tooth/implant position and surface.

**Figure 1:**
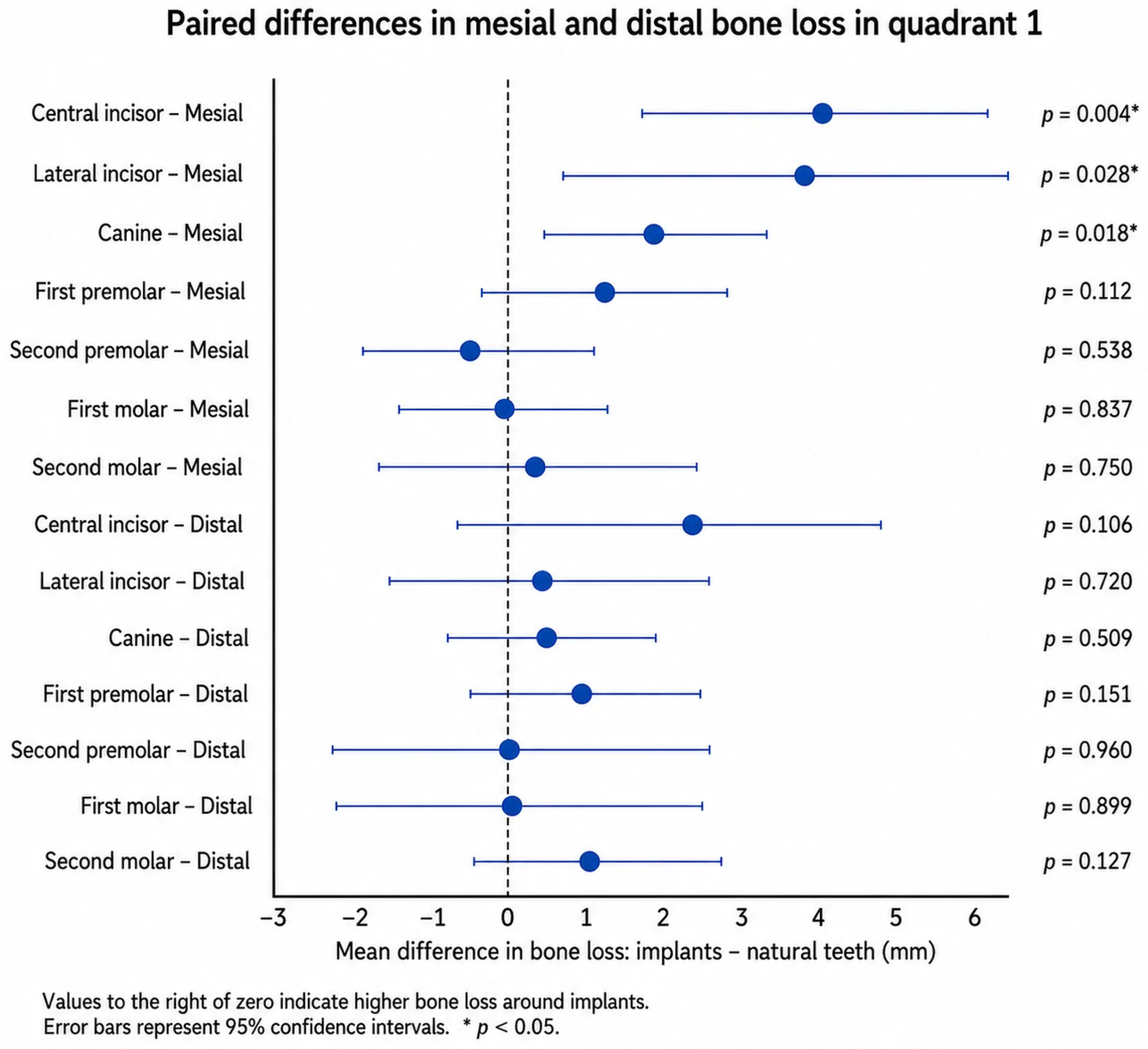
Forest plot of paired differences in mesial and distal bone loss between dental implants and natural teeth in quadrant 1. Positive values indicate greater bone loss around implants. Error bars represent 95% confidence intervals. Statistically significant differences were only observed for mesial surfaces in the anterior region.

The distribution of paired differences across anatomical positions is illustrated in Figure 1.

### 3.2. Mesial Bone Loss Comparisons Between Natural Teeth and Implants

The mesial surface showed the most relevant differences between natural teeth and implants. In the anterior region, mesial peri-implant bone loss was significantly greater than mesial periodontal bone loss around natural teeth. Specifically, significant differences were observed for the central incisor (4.90 ± 1.78 mm vs. 8.30 ± 3.31 mm; mean difference = 3.39 mm; t = 3.563; *p* = 0.004), lateral incisor (4.94 ± 2.82 mm vs. 8.12 ± 3.57 mm; mean difference = 3.18 mm; t = 2.477; *p* = 0.028), and canine (4.42 ± 1.26 mm vs. 5.99 ± 3.12 mm; mean difference = 1.57 mm; t = 2.558; *p* = 0.018).

In contrast, posterior positions did not show statistically significant mesial differences. Although the first premolar showed higher mesial bone loss around implants than around natural teeth (4.74 ± 2.81 mm vs. 5.89 ± 2.21 mm), the difference did not reach statistical significance (*p* = 0.112). Likewise, the second premolar, first molar, and second molar only showed small and statistically non-significant mesial differences.

**Figure 2:**
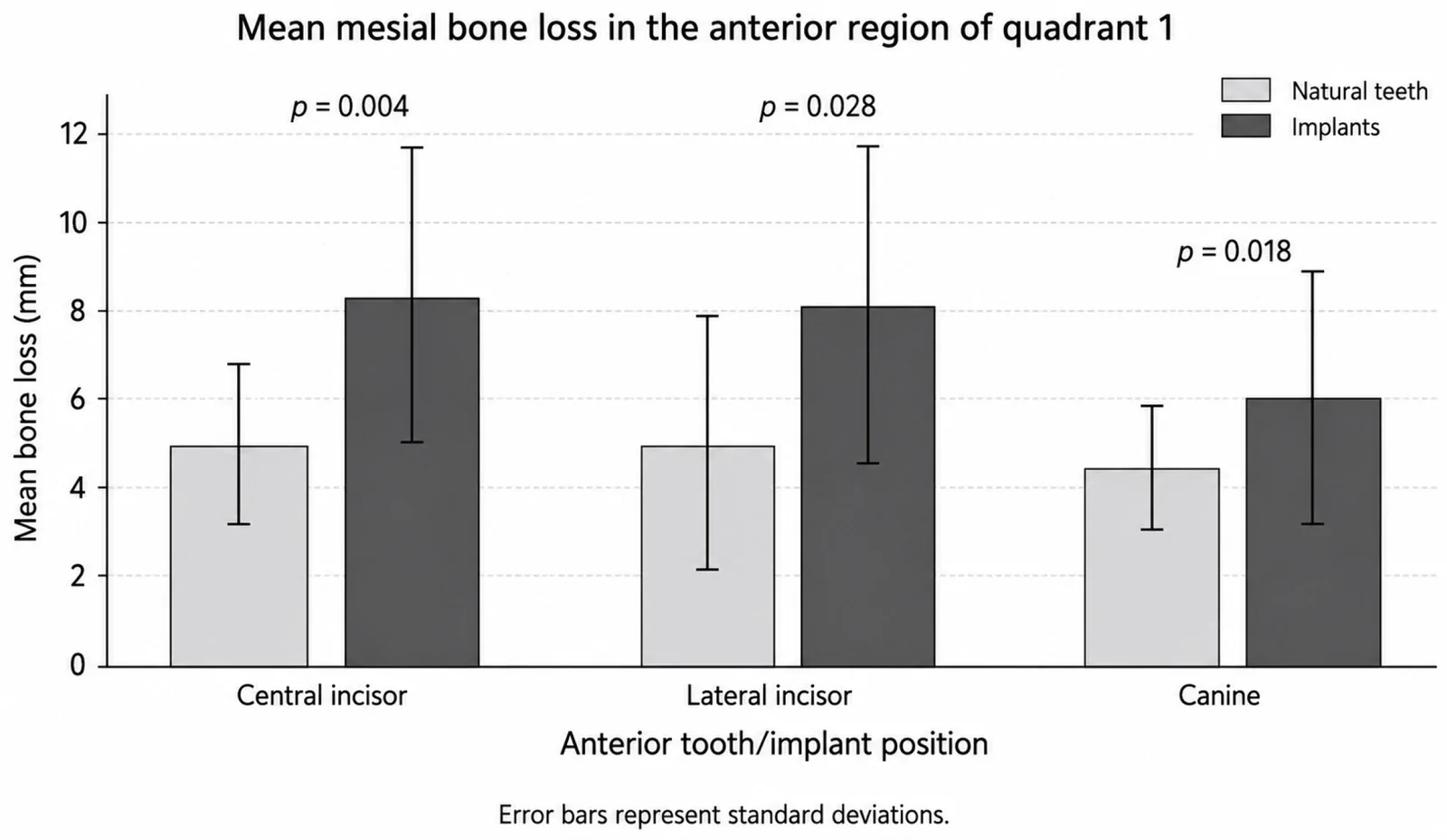
Mean mesial bone loss around natural teeth and implants in the anterior region of quadrant 1. Error bars represent standard deviations. Implants showed significantly greater mesial bone loss than natural teeth in the central incisor, lateral incisor, and canine positions.

Overall, these findings indicate that, in quadrant 1, mesial peri-implant bone loss was more pronounced than periodontal bone loss mainly in the anterior region. The comparative distribution of mean mesial bone loss in the anterior region is illustrated in Figure 2.

### 3.3. Distal Bone Loss Comparisons Between Natural Teeth and Implants

Distal comparisons showed a more balanced distribution of bone loss between natural teeth and implants. Although implants showed slightly higher distal mean values across several anatomical positions, none of the distal comparisons was statistically significant.

In the anterior region, distal bone loss was higher around implants in the central incisor, lateral incisor, and canine positions; however, these differences were not statistically significant. Similarly, no significant distal differences were observed in the posterior region, including premolars and molars. The largest distal posterior difference was observed for the second molar, but this comparison also remained non-significant.

Overall, distal bone loss appeared more comparable between natural teeth and implants than mesial bone loss, suggesting that the main site-specific difference was localized to the mesial surface rather than the distal surface.

### 3.4. Summary of Statistically Significant Findings

The statistically significant findings are summarized in Table 3.

Overall, the significant findings were confined to specific anterior mesial sites, supporting a localized rather than generalized pattern of radiographic bone loss differences between natural teeth and implants.

## 4. Discussion

The present study evaluated mesial and distal radiographic bone loss around natural teeth affected by periodontal disease and dental implants affected by peri-implantitis, with a site-specific approach focused on quadrant 1. The main finding was that peri-implant bone loss was significantly greater than periodontal bone loss only on the mesial surface of anterior implants, namely central incisors, lateral incisors, and canines. In contrast, distal surfaces and posterior tooth/implant positions did not show statistically significant differences. These findings suggest that peri-implant bone destruction may not simply reproduce the radiographic pattern of periodontal bone loss around natural teeth, but may instead show a surface and position-dependent distribution.

The observation that peri-implant bone loss was more pronounced in specific anterior mesial sites is clinically relevant. Previous studies have shown that peri-implantitis may progress in a non-linear and accelerating manner, with considerable variation between implants and patients [14,15,16]. This variability supports the need for site-specific radiographic assessment rather than only relying on global mean bone loss values. In the present analysis, the significant differences were not uniformly distributed across all surfaces or anatomical positions, but were concentrated in the anterior mesial region. This supports the hypothesis that peri-implant bone loss may be influenced by local anatomical, prosthetic, biomechanical, and hygiene-related factors.

The biological differences between natural teeth and dental implants may also explain the observed pattern. Natural teeth are supported by the periodontal ligament, cementum, and supracrestal connective tissue attachment, while implants are directly anchored to bone through osseointegration. Consequently, the inflammatory response and the progression of tissue destruction may differ between periodontal and peri-implant tissues. Consensus and review data indicate that peri-implantitis is characterized by inflammation in the peri-implant connective tissue associated with progressive supporting bone loss, and that its onset and progression may differ from periodontitis [6,10,17]. Therefore, the greater mesial bone loss observed around anterior implants in this study may reflect the distinct biological vulnerability of peri-implant tissues.

Radiographic assessment remains essential for monitoring both periodontal and peri-implant bone changes. In periodontitis, intraoral radiographs are commonly used to assess alveolar crest levels and interproximal bone loss [18,19]. Around implants, radiographic evaluation of marginal bone levels is central to diagnosis and follow-up, particularly when combined with clinical parameters such as probing depth, bleeding on probing, and suppuration [20,21]. The present study reinforces the value of separately evaluating mesial and distal surfaces because global or averaged measurements may obscure localized differences. In this dataset, the mesial surface was the primary site of significant differences, whereas distal surfaces were comparatively balanced.

The lack of statistically significant distal differences suggests that peri-implant and periodontal bone loss may be more comparable on distal surfaces than on mesial surfaces. Although implants frequently showed slightly higher distal mean values, these differences did not reach statistical significance. This finding is important because it indicates that the difference between periodontal and peri-implant bone destruction was not generalized across all interproximal surfaces. Instead, it was localized mainly to the mesial aspect of anterior implants. Similar site-dependent behavior has been described in the literature, where peri-implant marginal bone loss may vary according to implant position, prosthetic design, local anatomy, and maintenance conditions [22,23,24].

Several local factors may contribute to the mesial predominance observed in the anterior region. Implant position, emergence profile, prosthetic contour, accessibility for plaque control, and local tissue phenotype may influence biofilm accumulation and the stability of the peri-implant marginal bone [25,26]. In addition, occlusal loading and prosthetic design may affect peri-implant bone stress distribution, although the relationship between occlusal overload and peri-implant bone loss remains complex and not fully established [27,28]. In anterior areas, esthetic-driven implant placement and prosthetic emergence profiles may also influence access for hygiene and local inflammatory control. These factors may partially explain why mesial peri-implant bone loss was more evident in anterior sites.

The clinical implication of these findings is that anterior implants, particularly their mesial surfaces, may require careful radiographic monitoring during maintenance visits. Since peri-implantitis may progress rapidly and non-linearly in some cases, early identification of localized mesial bone changes may help clinicians intervene before more extensive circumferential bone loss develops [14,16]. The results also suggest that clinicians should avoid interpreting peri-implant bone loss as a uniform process. Instead, mesial and distal surfaces should be assessed independently, especially in anterior implant sites.

The present study has several limitations. First, the retrospective radiographic design limits causal interpretation. Although the study can identify site-specific radiographic patterns and associations, it cannot determine whether anatomical, prosthetic, occlusal, hygiene-related, or patient-related factors directly caused the observed mesial differences. Second, the analysis was limited to available paired radiographic observations from quadrant 1, and the number of paired sites varied according to tooth/implant position. Therefore, the findings should be interpreted as quadrant-specific and should not be automatically generalized to the entire dentition.

Third, several clinical and prosthetic parameters that may influence periodontal and peri-implant bone loss were not included in the present comparative model. These include probing depth, bleeding on probing, implant loading time, prosthetic design, emergence profile, prosthetic retention type, keratinized mucosa width, smoking status, systemic conditions, oral hygiene status, and maintenance history. These factors are known to influence the risk and progression of peri-implant disease and should be considered in future prospective studies [25,26,29].

Another limitation is related to the retrospective nature of CBCT assessment. Although CBCT provides three-dimensional information and allows for detailed evaluation of mesial and distal bone levels, radiographic measurements may be influenced by image quality, voxel size, patient positioning, artifacts, and section orientation. For this reason, standardized acquisition and measurement protocols remain essential for reliable interpretation of periodontal and peri-implant bone levels [19,30].

Finally, all CBCT measurements were performed by a single calibrated examiner. Therefore, inter-examiner reliability could not be assessed and this should be acknowledged as a methodological limitation. However, a subset of measurements was repeated by the same examiner after a two-week interval as an internal consistency check; no formal intra-examiner reliability coefficient was calculated. The analysis was restricted to maxillary quadrant 1, with relatively small subsample sizes ranging from 13 to 24 paired observations per anatomical position. These characteristics limit the statistical power of the position-specific comparisons and the generalizability of the findings beyond the investigated quadrant. Additionally, the use of different anatomical reference landmarks for natural teeth (cementoenamel junction) and implants (implant platform) may introduce structural measurement bias, as implant placement depth can vary with the surgical procedure. Therefore, direct comparisons of absolute bone-level measurements between teeth and implants should be interpreted with caution. Future studies should include multiple examiners, standardized calibration procedures, and formal intra- and inter-examiner reliability analysis to further strengthen measurement reproducibility.

Future studies should expand the analysis to all quadrants and include larger samples with standardized follow-up intervals. Additional variables such as implant system, implant diameter and length, loading time, prosthetic retention type, emergence profile, occlusal scheme, keratinized mucosa width, and periodontal history should be included in multivariable models. New digital tools, including automated or artificial-intelligence-assisted radiographic measurements, may also improve reproducibility and reduce observer-related variability in bone level assessment [16,31].

In summary, the present study suggests that peri-implant bone loss was only significantly greater than periodontal bone loss on the mesial surface of anterior implants in quadrant 1. Distal surfaces and posterior positions did not show significant differences. These findings support the concept that peri-implant bone destruction may have a site-specific radiographic pattern, rather than representing a simple equivalent of periodontal bone loss around natural teeth. A mesial–distal approach may therefore provide additional diagnostic value in the radiographic monitoring of periodontal and peri-implant disease.

## 5. Conclusions

The present study suggests that peri-implant bone loss may not uniformly reproduce the mesial–distal pattern of periodontal bone loss around natural teeth. Within the analyzed maxillary quadrant, differences were observed primarily on the mesial surfaces of anterior implants, particularly in the central incisor, lateral incisor, and canine regions, where greater radiographic bone loss was identified compared with the corresponding natural teeth.

Distal surfaces and posterior tooth/implant positions showed no statistically significant differences, indicating that the observed radiographic differences may be surface- and position-dependent. These findings highlight the potential clinical value of separately assessing mesial and distal bone levels during periodontal and peri-implant radiographic monitoring.

However, these findings should be interpreted cautiously in light of the retrospective, quadrant-specific design; the relatively small number of position-specific subsamples; and the clinical, prosthetic, and methodological factors that could not be fully controlled in the present radiographic analysis. Within these limitations, a mesial–distal assessment may provide additional insight into localized patterns of bone loss. Future prospective studies with larger samples, inclusion of all dental quadrants, and comprehensive clinical and prosthetic characterization are needed to confirm these observations and further clarify site-specific peri-implant bone loss patterns.

## Figures and Tables

**Table 1:** Demographic and clinical characteristics of the study population.

Characteristic	Value
Number of patients	128
Inclusion period	January 2024–December 2025
Age range, years	45–67
Women, n (%)	87 (68.0%)
Men, n (%)	41 (32.0%)
Imaging modality	CBCT (cone-beam computed tomography)
Diagnosis around natural teeth	Periodontitis with alveolar bone loss
Diagnosis around implants	Peri-implantitis with peri-implant bone loss
Analyzed quadrant	Quadrant 1
Analyzed surfaces	Mesial and distal
Main measured variable	Bone loss, mm

**Table 2:** Paired comparison of mesial and distal bone loss between natural teeth and implants in quadrant 1.

Tooth/Implant Position	Surface	Natural Teeth, Mean ± SD (mm)	Implants, Mean ± SD (mm)	Mean Difference, Implants − Natural Teeth	t	df	*p*-Value
Central incisor	Mesial	4.90 ± 1.78	8.30 ± 3.31	3.39	3.563	12	0.004
Central incisor	Distal	4.50 ± 1.85	6.58 ± 3.32	2.08	1.745	12	0.106
Lateral incisor	Mesial	4.94 ± 2.82	8.12 ± 3.57	3.18	2.477	13	0.028
Lateral incisor	Distal	4.58 ± 2.04	4.93 ± 2.75	0.35	0.366	13	0.720
Canine	Mesial	4.42 ± 1.26	5.99 ± 3.12	1.57	2.558	21	0.018
Canine	Distal	5.43 ± 2.80	5.89 ± 2.30	0.46	0.672	21	0.509
First premolar	Mesial	4.74 ± 2.81	5.89 ± 2.21	1.15	1.655	23	0.112
First premolar	Distal	4.31 ± 2.44	5.34 ± 1.92	1.03	1.484	23	0.151
Second premolar	Mesial	4.92 ± 2.08	4.50 ± 1.72	−0.42	−0.626	20	0.538
Second premolar	Distal	5.49 ± 3.12	5.55 ± 4.20	0.06	0.050	20	0.960
First molar	Mesial	5.10 ± 2.28	4.98 ± 1.99	−0.12	−0.210	14	0.837
First molar	Distal	4.96 ± 3.15	5.10 ± 1.69	0.14	0.130	14	0.899
Second molar	Mesial	5.69 ± 3.35	6.01 ± 2.15	0.32	0.323	18	0.750
Second molar	Distal	5.32 ± 2.22	6.45 ± 1.92	1.13	1.601	18	0.127

Note: Positive mean differences indicate greater bone loss around implants than around natural teeth, whereas negative values indicate greater bone loss around natural teeth.

**Table 3:** Summary of statistically significant findings in quadrant 1.

Region	Surface	Significant Comparisons	Interpretation
Anterior	Mesial	Central incisor, *p* = 0.004; lateral incisor, *p* = 0.028; canine, *p* = 0.018	Greater bone loss around implants
Anterior	Distal	None	Comparable distal bone loss
Posterior	Mesial	None	Comparable posterior mesial values
Posterior	Distal	None	Comparable posterior distal values

## Data Availability

The data supporting the findings of this study are available from the corresponding authors upon reasonable request. The data are not publicly available due to privacy and ethical considerations related to the retrospective use of patient-derived radiographic data.
